# *Infectious Agents and Cancer* reviewer acknowledgement 2015

**DOI:** 10.1186/s13027-016-0055-z

**Published:** 2016-02-10

**Authors:** Franco M. Buonaguro, Sam M. Mbulaiteye, Maria Lina Tornesello

**Affiliations:** Istituto Nazionale Tumori, Naples, Italy; National Cancer Institute, Bethesda, USA; Istituto Nazionale Tumori, Naples, Italy

## Abstract

The editors of *Infectious Agents and Cancer* would like to thank all our reviewers who have contributed their time and expertise to the journal in Volume 10 (2015).

**Sayed Abdelwahab**

Egypt

**Ali Abdualsalam**

Yemen

**Amit Achhra**

Australia

**Clement Adebamovo**

USA

**Francisco Aguayo**

Chile

**Kayode Ajenifuja**

Nigeria

**Vidya Ajila**

India

**Elena Allia**

Italy

**Clorinda Annunziata**

Italy

**Ines Badano**

Argentina

**Iacopo Baussano**

France

**Robert Biggar**

USA

**Sabrina Bimonte**

Italy

**Julia Bohlius**

Switzerland

**Patrizia Bonelli**

Italy

**Álvaro H Borges**

Denmark

**Idris Brima**

Sudan

**Franco Buonaguro**

Italy

**Monica Cantile**

Italy

**Francesca Carozzi**

Italy

**Flair J Carrilho**

Brazil

**Sekar Pedamallu Chandra**

USA

**Kimon Chatzistamatiou**

Greece

**Carla Chibwesha**

South Africa

**Admire Chikandiw**

South Africa

**Lucy Chimoyi**

South Africa

**Nyasha Chin'Ombe**

Zimbabwe

**Roberto Cianni**

Italy

**Anna Coghil**

USA

**Manola Comar**

Italy

**Gaetano Corazzelli**

Italy

**Silvia De Sanjose**

Spain

**Annarosa Del Mistro**

Germany

**Katarzyna Derwich**

Poland

**Paola Di Bonito**

Italy

**Juan Du**

United States

**Ola Forslund**

Sweden

**Renato Franco**

Italy

**Aharon Freud**

USA

**E. M. Fuentes-Panama**

Mexico

**Franco Fulciniti**

Switzerland

**Marisa Gariglio**

Italy

**Paolo Giorgi Rossi**

Italy

**Lucia Giovannelli**

Italy

**Paolo Giovenali**

Italy

**Patricia Goggin**

Canada

**Shalaka Hampras**

USA

**Maria Hatziapostolou**

USA

**Andrew M Hill**

UK

**Dieter Hoffmann**

Germany

**Jin Huang**

China

**Tetsuya Ikeda**

Japan

**Somayeh Jalilvand**

Iran

**Phyllis J Kanki**

USA

**Shyam Kottilil**

USA

**Mineo Kurokawa**

Japan

**Anton Kutikhin**

UK

**Ke Lan**

China

**Wen Li**

China

**Xingnan Li**

USA

**Flavia Lillo**

Italy

**Morgan Marks**

USA

**Koji Matsumoto**

Japan

**Sam Mbulaiteye**

USA

**Alicia Mcdonald**

USA

**L Menezes**

Brazil

**J Mosmann**

Argentina

**Emmanuel Mugisha**

Uganda

**P G Murray**

UK

**Jonah Musa**

Nigeria

**Luciano Mutti**

UK

**Miriam Nakalembe**

Uganda

**Jacques Neefjes**

Netherlands

**Twalib Ngoma**

Tanzania

**Souichi Nukuzuma**

Japan

**Josè Oteo**

Spain

**Ridha Oueslati**

Tunisia

**Giuseppe Palma**

Italy

**H R Park**

South Korea

**Francesca Pentimalli**

Italy

**Annacarmen Petrizzo**

Italy

**Francesca Pezzuto**

Italy

**Eric Piaton**

France

**Maurizio Provenzano**

Italy

**Fabiana Quaglia**

Italy

**Camille Ragin**

USA

**Yasir Raza**

Pakistan

**Matejka Rebolj**

Denmark

**Milan Reinis**

Czech Republic

**Chad Ritch**

USA

**Frank Romeo**

Italy

**Salvatore Ronsini**

Italy

**Giovanni Battista Rossi**

Italy

**Martha-Eugenia Ruiz-Tachiquin**

Mexico

**Adolfo G. Sadile**

Italy

**Pierre Saintigny**

France

**Martina Salakova**

Czech Republic

**Mauricio Salcedo**

Mexico

**Omer Salim**

Sudan

**Sergio Sandrucci**

Italy

**Rodrigo Silvestre**

Brazil

**Kenneth Simbiri**

USA

**Freddy Sitas**

Australia

**Leon Snyman**

South Africa

**Nema Soliman**

Egypt

**Cheng Song**

China

**Madeleine Sporleder**

Germany

**Jatupol Srisomboon**

Thailand

**Noemy Starita**

Italy

**Giovanni Stellato**

Italy

**Caecilia Hapsari Ceriapuri Sukowati**

Italy

**Martins Tanimola**

UK

**Mauro Tognon**

Italy

**Maria Lina Tornesello**

Italy

**Ines Tozetti**

Brazil

**M Trimech**

France

**Piamkamon Vacharotayangul**

Thailand

**Hernán Vargas**

Colombia

**Aldo Venuti**

Italy

**Fernanda Visioli**

Brazil

**Monika Wagner**

Canada

**Thomas Weiss**

USA

**Denise Whitby**

USA

**Xin Xu**

USA

**Geng Yu**

USA

**Sonia Zapata**

Ecuador

**Apostolos Zaravinos**

Sweden

**Huachun Zou**

Australia

**Antonella Zucchetto**

Italy

